# Supplementary material for: Identification of Freezing-Responsive microRNAs and Their Targets in Chinese Jujube by Small RNA and Degradome Sequencing
Source: Int J Mol Sci. 2026 Sep 4;27(17):7912. doi: 10.3390/ijms27177912 (PMC13566890; doi:10.3390/ijms27177912)
Supplement: Supplementary file 1 [file ijms-27-07912-s001.zip › Supplementary Material S2/novel_miR_69.pdf]

Provisional ID	:	novel_miR_69
Score total	:	0.5
Score for star read(s)	:	-1.3
Score for read counts	:	0
Score for mfe	:	0.2
Score for randfold	:	1.6
Score for cons. seed	:	
Total read count	:	48
Mature read count	:	48
Loop read count	:	0
Star read count	:	0

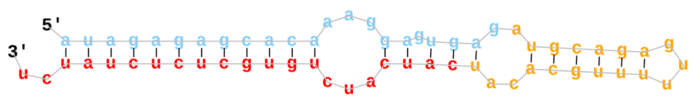

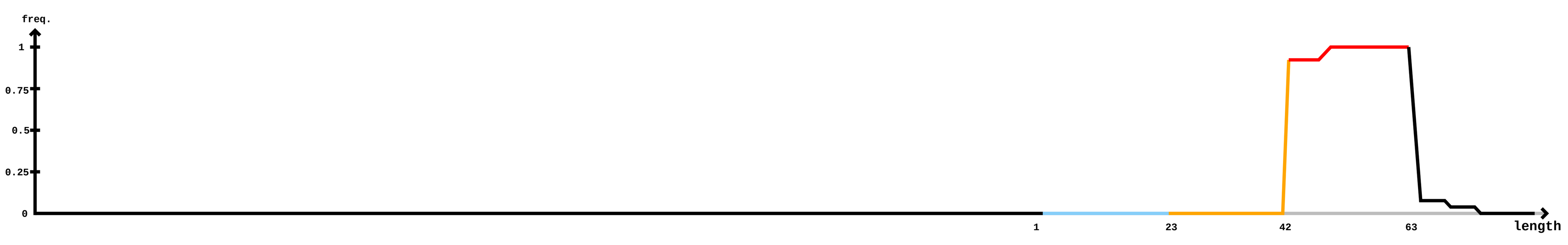

Star Mature

```
5'- uaaugauaaugugaaauuugggaggaaaauuaaagaaaauaaaccaguaaauauuaucuaaaccauggaaauuaaaaauuaaagaacuaauacaauauuguauaauauaaguaaaaguugauaugaauaauagagagggugggggcuuaaaugugauggcagaagcauagagagcacaaaggagugagaugcagaguuuuugcacaucaucaucugugcucucuaucuucugucaacaacauuaaucaau -3'   exp
    ....((((((((.....((((......................))))...))))))))....((((...........(((...((((..(((.(((((....)))))..)))..))))....)))...........)))).......(((((.(((((((((.((((((((((((((...((.(((..((((((...))))))..)))))...)))))))))))))))))))))).).)))))....... reads   mm   sample
    ...............................................................................................................................................................................................................cCucaucugugcucucuaucu...................... 1   1   LCK-2
    ...............................................................................................................................................................................................................caucaucugugcucucuaucu...................... 24   0   LCK-1
    ...............................................................................................................................................................................................................caucaucugugcucucuaucu...................... 23   0   LCK-2
    .....................................................................................................................................................................................................................Uugugcucucuaucuucuguc................ 2   1   LT-2
    ......................................................................................................................................................................................................................ugugcucucuaucuucugucaUcaa........... 2   1   JCK-2
```
